# Condensate loss estimation and transient solar-to-vapor conversion efficiency for effective performance evaluation of the inclined solar still

**DOI:** 10.1016/j.mex.2022.101837

**Published:** 2022-08-29

**Authors:** Tijani Oladoyin Abimbola, Husna Takaijudin, Balbir Singh Mahinder Singh, Khamaruzaman Wan Yusof, Abdurrasheed Said Abdurrasheed, Ebrahim Hamid Hussein Al-Qadami, Abubakar Sadiq Isah, Kai Xian Wong, Nur Farhanah Ahmad Nadzri, Samiat Abike Ishola, Suleiman Akilu, Tunji Adetayo Owoseni

**Affiliations:** a Department of Civil and Environmental Engineering, Universiti Teknologi PETRONAS, 32610 Bandar Seri Iskandar, Perak, Malaysia; b Department of Fundamental and Applied Sciences, Universiti Teknologi PETRONAS, 32610 Bandar Seri Iskandar, Perak, Malaysia; c Department of Civil Engineering, Ahmadu Bello University, Zaria, Kaduna State, Nigeria; d Department of Civil and Environmental Engineering, University of Nottingham, Semenyih 43500, Selangor, Malaysia; e Department of Marine Sciences, University of Lagos, Akoka, Lagos State, Nigeria; f Centre for Nanotechnology Research, Universiti Teknologi PETRONAS, 32610 Bandar Seri Iskandar, Perak, Malaysia; g Department of Mechanical Engineering, the University of Nottingham, NG72RD, United Kingdom

**Keywords:** Solar still, Vaporization, Condensation, Solar-thermal energy, Latent heat, Solar-to-vapor conversion

## Abstract

Previously, freshwater yields of the solar still were quantified only based on the actual distillate recovery, not considering condensate losses by any means. Likewise, solar-to-vapor conversion efficiencies of the solar still were conventionally considered and evaluated as one-off -rigid values- based on the latent heat of the average water temperature. In most cases, these approaches do not give a comprehensive performance details of the solar still. Thus, we suggest two considerations for effective performance evaluation of the inclined solar still. The first consideration is theoretical estimation of the condensate loss due to the condensate collection channel slope, while the other is the use of a transient method to evaluate the solar-to-vapor conversion efficiency. We demonstrated, geometrically, that the condensate loss on the inclined solar still can be significant—hence the need to consider it alongside the overall yield. We formulated a model to estimate the condensate loss and validated the model by comparing an estimated condensate loss with experimental loss. Similarly, we demonstrated a transient approach to evaluate the solar-to-vapor conversion efficiency by using the latent heat of the hourly water temperature. Accordingly, the optimum hourly efficiency of the investigated solar still prototype was 161.4%, with a daily average of 113.4% versus 108.4% from the conventional method. Overall, no study on the solar still had previously accounted for condensate losses by any means whatsoever, making our current study a reference and a pioneer in this concept and suggesting an advancement in the approach to report the performance productivity of the solar still.

• Condensate loss on the inclined solar still due condensate collection channel slope was estimated geometrically and demonstrated to be significant.
• Solar-to-vapor conversion efficiency was evaluated using an hourly transient approach.

## Graphical abstract

**Figure:**
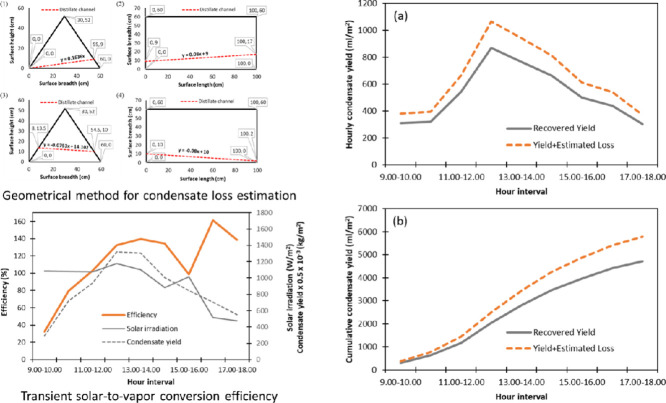

Specifications table

**Table:** 

Subject Area:	*Environmental Science*
More specific subject area:	*Desalination and water treatment*
Method name:	• *Geometrical method for condensate loss estimation in the inclined solar still.* • *Transient evaluation of solar-to-vapor conversion efficiency in solar still.*
Name and reference of original method:	*Potable water production using the solar still*[10,^11^,14]
Resource availability:	*Comprehensive passive-mode performance analysis on a new multiple-mode solar still for sustainable clean water processing*[2]

## Study background

Although the higher fraction of the earthbound water is saline, the global renewable freshwater reserve -amounting to as huge as 45.5-tera ton/year- is enough to cater for the entire human needs. However, the uneven distribution of the freshwater resources by nature is a principal natural factor that has led to water scarcity at the deprived regions [6]. Differently, population growth, industrialization and pollution dispersal, are amongst the man-made factors that have enhanced water shortage where freshwater resources exist [7]. Addressing both the natural and the man-induced water scarcity, studies on engineering solutions, involving desalination and water reuse to augment freshwater supply, are on the rise [9]. In that respect, the solar still technology targets the remote and low-income settlements for clean water processing [3], while various solar still prototypes have been invented, fabricated and tested.

### The solar still

**Fig. 1:**
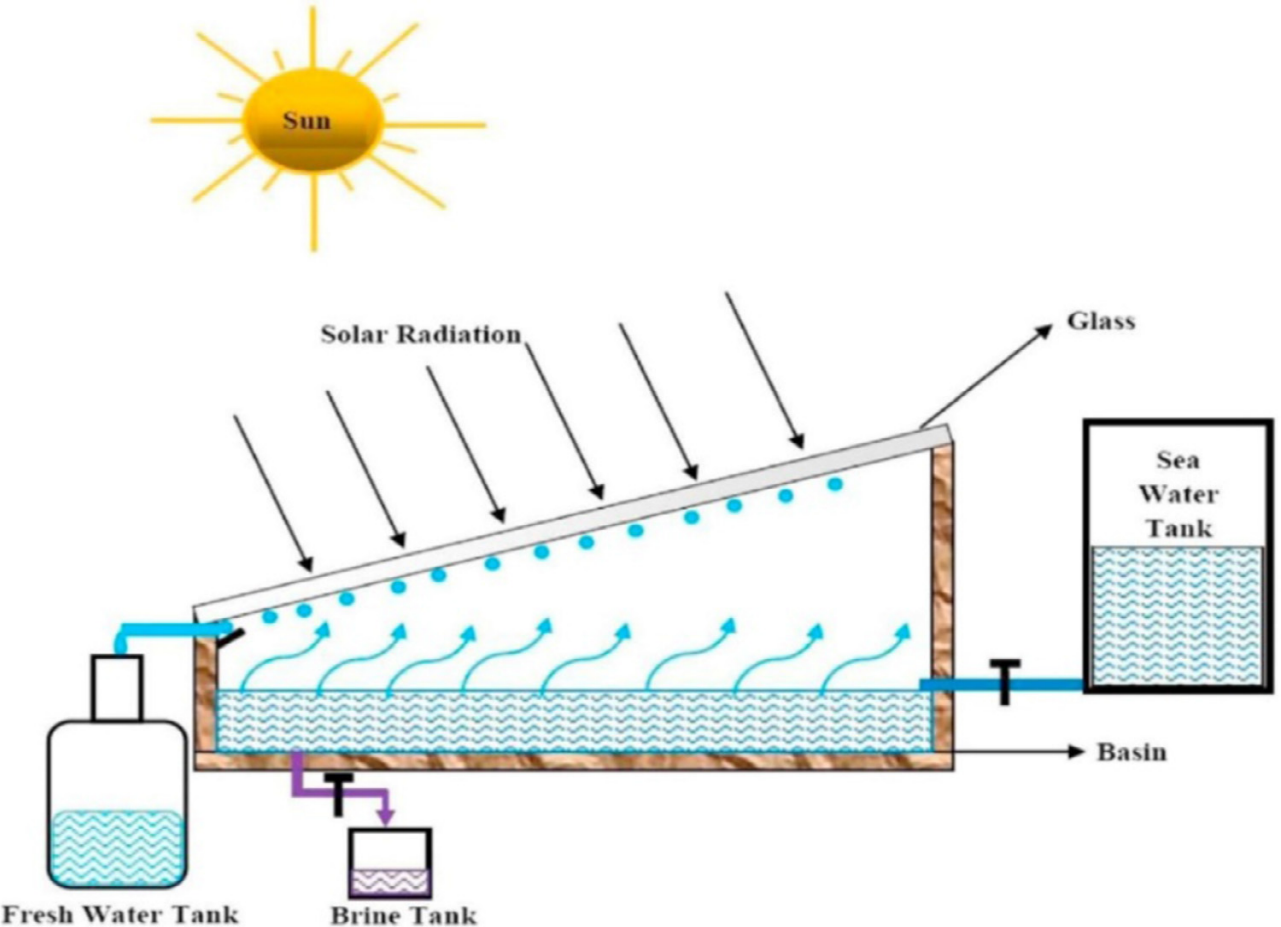
Freshwater extraction process of the solar still [15].

In function, the solar still is a device which predominantly utilizes solar radiation to extract clean potable water from unclean or contaminated water through simple evaporation and condensation processes. In design, the solar still is a closed-system assembly, which comprises mainly a flat metal basin, an inclined transparent glass cover and a bottom and side insulation. As Fig. 1 illustrates, when the metal basin is fed with raw water from an unclean water source and the system is expose to sunlight, the basin -being a good conductor of heat- absorbs solar radiation to initiate a heat-and-mass transfer phenomenon through which the raw water gradually evaporates. Then, the resultant vapour -in contact with the glass cover- condenses to produce freshwater.

The output performance of the solar still is usually measured in two ways. The first is by its *productivity*, which is the amount of the condensate collected as distilled water per basin square meter. Using this method, the condensate on the wall of the glass cover is collected through installed drainage channels and measured as volume or mass. The other way is by its *solar-to-vapor efficiency*, which is the magnitude of the solar radiation that is converted to vapor per basin square meter. This is done by comparing the mass of the distillate collected from the solar still to the magnitude of the solar radiation received by the basin, while assuming the solar still to be a closed system.

### Method details

In evaluating the performance of the solar still, losses in condensate due to the condensate collection channel slope in the double slope solar still were not accounted for. Similarly, the solar-to-vapor conversion efficiencies were only evaluated based on the daily average temperature values, rather than based on the transient hourly average temperature. These two limitations suggest that the conventional methods for the performance evaluation of the solar still, regarding the freshwater productivity and the solar-to-vapor conversion efficiency, do not entirely indicate the actual output of the device. Therefore, in this study, we propose two considerations for a more comprehensive performance evaluation of the double-slope inclined solar still. The first consideration is a geometrical method to factor-in the condensate loss in the overall distillate productivity, while the other is the use of the actual transient hourly temperature values for the evaluation of the solar-to-vapor conversion efficiency as a replacement for the fixed daily average temperature—thus, giving rise to a transient solar-to-vapor conversion efficiency.

Although these two considerations aim at improving the overall method of evaluating the performance of the solar still, the condensate loss estimation targets to account for the unrecovered condensate during solar still experiments owing to the slope of the condensate channel, while the transient solar-to-vapor conversion efficiency approach targets to simplify the efficiency of the solar still on hourly basis rather than on usual daily basis. To validate the two considerations, the condensate loss on a double-slope solar still prototype was calculated geometrically and compared with the actual experimental losses, while on the other hand, the average solar-to-vapour conversion efficiency computed both on the daily and the hourly bases were also compared.

1. A geometrical method for condensate loss estimation

In the double-slope inclined solar still, the inclination of the glass cover is crucial in many ways to enhance productivity of the device. The specific angle of inclination of the glass cover dictates the effectiveness of solar radiation absorptivity by the water-containing basin, while the resulting vapor from the water entirely relies on the glass cover to condense [4,13]. By and large, our consideration for the glass cover in this study is restricted only to its function as a condensation surface, which can be summarized into two, viz.:

a. As a barrier between the enclosed vapor molecules at the inner-side of the solar still and the outer ambient atmosphere, the glass cover serves as a boundary for heat-exchange between the vapor and the external ambience. Eventually, the vapor cools and condenses on the inner surface of the glass due to temperature difference across the boundary's inner and outer layers.
b. After gaining enough momentum, the condensate falls through the glass inclination into a length of condensate collection channel lined along the base of the glass and drains through the channel slope into a main collection vessel as clean water.

In practice, to ensure effective drainage of condensate off the channel, as mentioned in (b), there is need to incline the channel at a specific design slope reference to the horizontal. To achieve the channel slope requirement, a fraction of the condensation surface area is traded-off, eventually—and by implication, only the condensate above the channel are recoverable as distillate, while the fraction below the channel are regarded as losses.

Previous method to quantify the distillate output of this type of solar still does not account for the losses alongside the overall productivity [5,10,11,14,16,17]; the results from which do not entirely suggest the actual freshwater yield of the solar still, especially when the area below the channel is significant. Therefore, the need to factor-in the losses in the overall output of a solar still is crucial, especially to enhance subsequent system upgrade and to make adequate comparison between a solar still prototype and another. We hereby introduce and demonstrate in our study a theoretical method to analyse and capture the amount of condensate loss due to the channel slope using geometry.

### Method assumptions and procedure

For replicability, it is assumed for the use of our geometrical method for condensate loss estimation in an inclined solar still that the solar still is a closed system and that all the surfaces of the condensation glass cover are made of the same material and have the same condensation pattern. Thus, the procedures for the method are highlighted as follows:

i. The total area of each condensation surface $\left(CS_{i}\right)$ of the glass cover where vapour condensation occurs is calculated based on the shape of the surface as: $CS_{1}$, $CS_{2}$,…, $CS_{n}$.
ii. The specific area of the portion of each condensation surface $\partial CS_{i}$ where condensate loss occurs is calculated based on the shape of such portion as: $\partial CS_{1}$, $\partial CS_{2}$, …, $\partial CS_{n}$.
iii. The condensate loss fraction at each condensation surface is evaluated as: $\partial CS_{1}\;/\;CS_{1}$, $\partial CS_{2}\;/\;CS_{2}$, …, $\partial CS_{n}\;/\;CS_{n}$ to compare condensate loss at each surface.
iv. Whereas the overall condensate loss fraction $\left(\delta_{loss}\right)$ for all the surfaces is evaluated as:
  (1) $$\delta_{loss}\;=\;\sum_{i=1}^{n}\partial CS_{i}\;/\;\sum_{i=1}^{n}CS_{i}$$
v. Then, the total recoverable condensate (Q) from the solar still will be evaluated in terms of the actual collected condensate (m) and the estimated loss fraction $\left(\delta_{loss}\right)$ as:
  (2) $$Q=\;m\;/\;\left(1-\delta_{loss}\right)$$
vi. Finally, the actual condensate loss $\left(m_{loss}\right)$ will be calculated in terms of the Q and $\delta_{loss}$ as:
  (3) $$m_{loss}=Q.\delta_{loss}$$
2. Transient solar-to-vapor conversion efficiency

The solar still benefits from the solar irradiation to convert water to vapor. The extent to which the solar still leverages the incident solar irradiation to vaporize water is known as the solar-to-vapor conversion efficiency. In other words, the efficiency of a solar still does not depend on the solar radiation itself. It rather depends on how much of the irradiance is absorbed by the basin of the solar still, retained and utilized to form water vapor [2]. Thus, the evaluation of the solar-to-vapor conversion efficiency is important to comprehensively describe the performance of a solar still unit.

Previous studies [1,8,10,12,16,18,20] have evaluated the efficiency on the basis of the average temperature-dependent latent heat of vaporization of the feed water and the average irradiance received by the basin across the productivity hours, as giving in Eq. (4) and usually referred to as *daily efficiency* ($\eta_{sv}$), where $\dot{m}$ is the hourly distillate yield, in kg; $A_{s}$ is the surface area of the basin, in m^2^; $I_{\left(t\right)}$ is the hourly irradiance, in W/m^2^; and $h_{fg}$ is the latent heat of vaporization of water, in J/kg.

$h_{fg}$ is dependent on the water temperature and can be obtained directly from tables of thermodynamic properties of water at the corresponding temperature value or otherwise calculated using Eq. (5) [12,19], where $T_{w}$ is the average water temperature across the productivity hours.

(4) $$\eta_{sv}=\;\frac{\sum \dot{m}\;\times h_{fg}}{A_{s}\times \;\sum I_{\left(t\right)}}$$

(5) $$h_{fg}=2.493\;\times 10^{6}\left[1-\left(9.4479\;\times 10^{-4}T_{w}+1.3132\;\times 10^{-7}T_{w}^{2}-4.794\;\times 10^{-9}T_{w}^{3}\right)\right]$$

However, instead of the conventional approach, which is based on daily average values of the variables, we consider the use of instantaneous hourly value of each variable to arrive at *transient efficiency* ($\eta_{sv-trans}$), which is the solar-to-vapor conversion efficiency of the solar still at each productivity hour.

### Method procedure

i. The efficiency of the solar still is calculated as transient values on hourly bases as described by Eq. (6), where all variables remain as previously defined. Whereas $h_{fg}$ in this case can be calculated using Eq. (7), where $T_{wi}$ is the instantaneous hourly temperature of the feed water. Accordingly, the average or daily efficiency, if required, can then be evaluated from the transient values by using Eq. (8), where n is the number of productivity hours.
  (6) $$\eta_{sv-trans}=\;\frac{\dot{m}\;\times h_{fg}}{A_{s}\times I_{\left(t\right)}}$$
  (7) $$h_{fg}=2.493\;\times 10^{6}\left[1-\left(9.4479\;\times 10^{-4}T_{wi}+1.3132\;\times 10^{-7}T_{wi}^{2}-4.794\;\times 10^{-9}T_{wi}^{3}\right)\right]$$
ii. The average efficiency is then computed from the hourly values to get the daily average efficiency using Eq. (8), where n is the number of experimental hours.
  (8) $${\bar{X}}_{\eta }=\frac{\sum_{i=1}^{n}\left[\frac{\dot{m}\;\times h_{fg}}{A_{s}\times I_{\left(t\right)}}\right]}{n}$$

This approach is logical because the water temperature is dynamic and transient from one productivity hour to another, vis-à-vis the enthalpy of vaporization. Also, the difference between the lowest and the highest temperature values could be highly significant, for which an average value will not truly represent the temperature at each hour [2].

## Method demonstration and validation

**Fig. 2:**
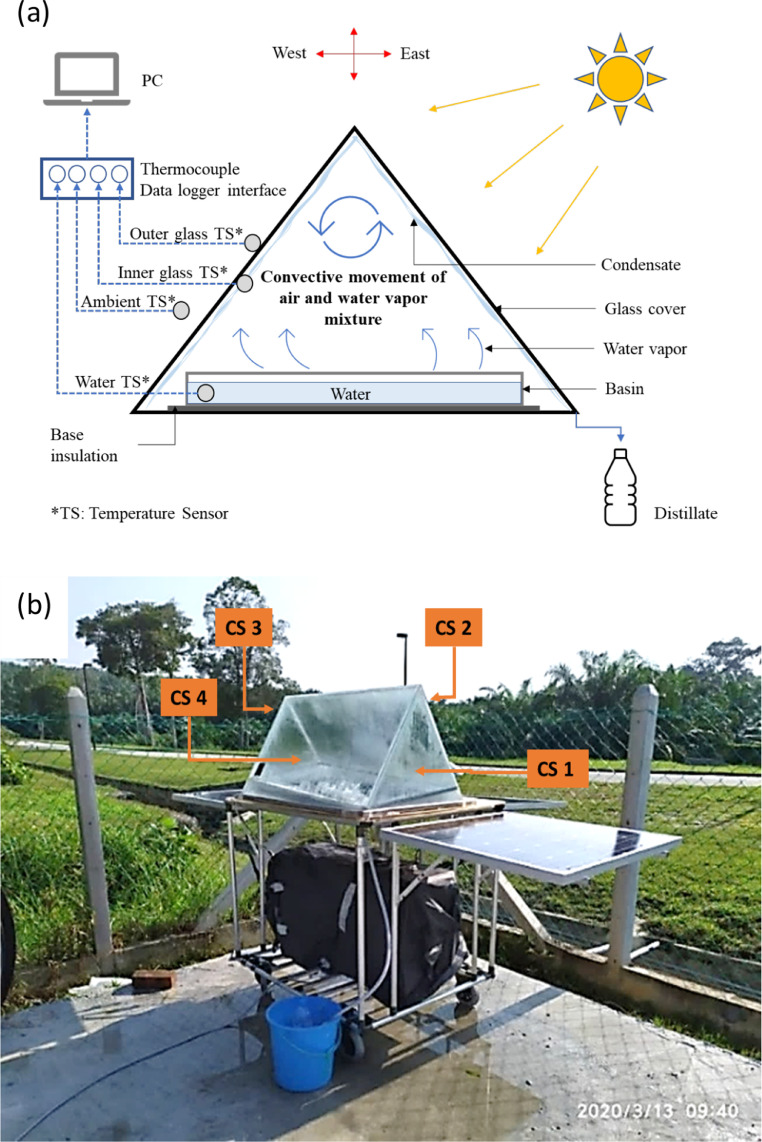
Experimental setup for the investigated solar still prototype showing (a) the experimental schematic and (b) actual photograph of the solar still during the experiment.

We demonstrated the two aforementioned considerations in our original study [2] to estimate the average condensate yield and the optimum solar-to-vapor conversion efficiency of our solar still prototype. While details of the solar still prototype and the comprehensive experimental procedures and operating conditions are contained in the original article, Fig. 2 shows the experimental setup, where Fig. 2-a is the schematic of the experimental setup and Fig. 2-b is a photograph of the solar still during the actual experiment. We conducted desalination investigations on the solar still in the passive mode, relying entirely on direct insolation. For each experiment, the actual condensate yields were collected hourly between 9:00 and 16:00 hours for 3 days. The cumulative experimental loss was collected only after the end of each experimental day, while the theoretical condensate loss was estimated. Similarly, the transient solar-to-vapor conversion efficiency, which is a measure of how well the solar still leveraged the incident solar irradiation to produce water vapor on hourly basis, were evaluated using Eq. 6. The underlying paragraphs summarize our findings.

1. *Geometrical condensate loss estimation*

**Fig. 3:**
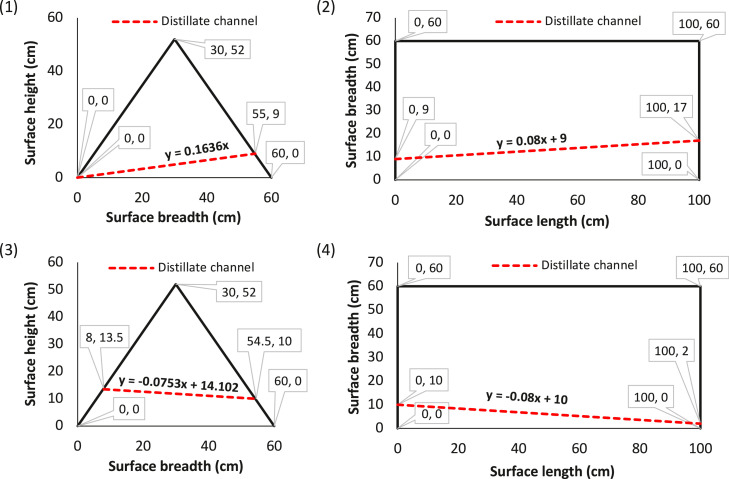
Perimeter coordinate plots of the condensation surfaces 1-4 of the solar still showing the distillate channels and their corresponding slopes.

While the full experimental hourly yield of condensate is attached in Appendix A, Table 1 shows the cumulative experimental yields from the solar still during a 3-day trials of seawater desalination and the cumulative recovered loss after each experimental day. The cumulative average experimental yield was 3842.2 ml/m^2^, while the cumulative average experimentally recovered loss was 657.9 ml/m^2^. On the other hand, the total condensate loss from the condensation surfaces (CS 1-4) due to the channel slope was estimated theoretically to be compared with the experimental loss. Fig. 3 shows the perimeter plots of the four condensation surface areas of the glass-cover of the solar still. The condensate collection channel at each of the four surfaces are indicated with the dotted lines, while the corresponding slope of each channel is shown with the straight-line equations. From Table 2, the designed condensation surface area and the total area of condensate loss at the surface were 15120 and 2797.9 cm^2^, respectively. Therefore, the overall condensate loss fraction $\left(\delta_{loss}\right)$from all the condensation surfaces estimated using Eq. 1 amounted to 18.5% of the total condensation surface area. Further, the total average recoverable condensate (Q) from the solar still within the experimental interval was calculated using Eq. 2, from which the theoretical condensate loss $\left(m_{loss}\right)$ was evaluated using Eq. 3 as 832.6 ml/m^2^. Comparison between the experimental loss and estimated theoretical loss at 15% degree of freedom gives a coefficient of variation of 0.117, which validates the suggested model.

**Table 1:** Experimental condensate yield (m) and recovered loss $\left(m_{loss}\right)$, in ml/m^2^, from 3-days seawater desalination experiment.

Trials	Day 1		Day 2		Day 3		Average	
	m	$m_{loss}$	m	$m_{loss}$	m	$m_{loss}$	m	$m_{loss}$
Cumulative yield	3229.6	529.7	3587.7	595.6	4711.1	815.0	3842.8	657.9

**Table 2:** Condensate loss estimation at the condensation surfaces 1-4 of the solar still.

Surface	Area of surface (cm^2^)	Area of condensate loss at surface (cm^2^)	Condensate loss at surface (%)	Contribution of surface to overall condensate loss (%)
1	1560.0	270.0	17.3	9.7
2	6000.0	1300.0	21.7	46.5
3	1560.0	627.9	40.2	22.4
4	6000.0	600.0	10.0	21.4

**Fig. 4:**
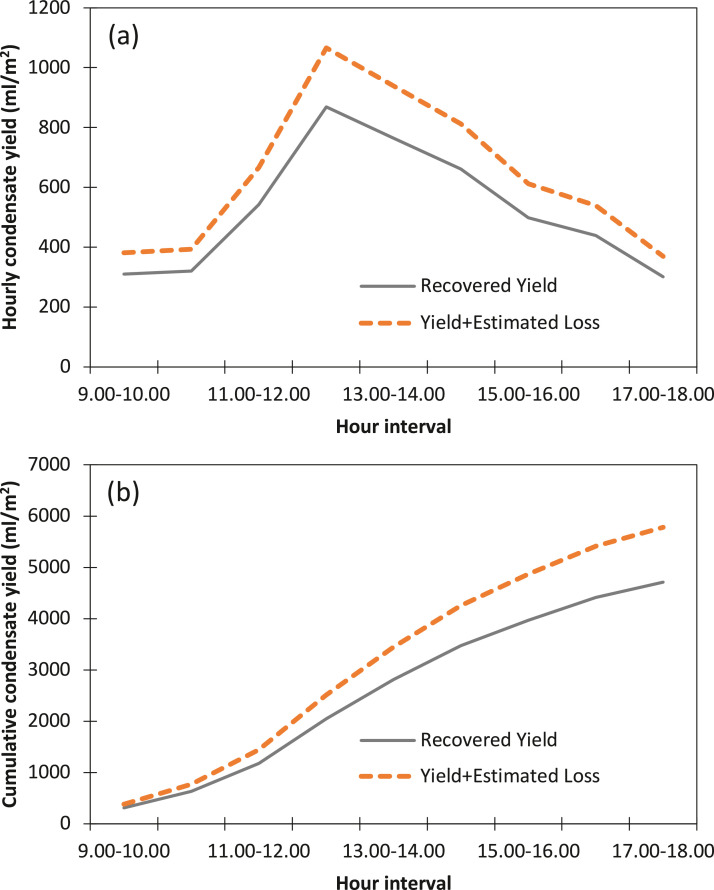
(a) hourly and (b) cumulative comparisons between actual recovered yield and yield with estimated-loss.

To demonstrate the significance of the condensate loss estimation on the solar still condensate yield evaluation, comparisons are made between the conventional yield measurement method, which does not include the loss factor, and our introduced consideration of an inclusion of the estimated loss. Fig. 4 compares the actual recovered yield of the optimum outcome i.e., Day 3 of our experimental trials with the estimated loss-included yield. The highest hourly recovered yield (Fig. 3-a) was 869.1 ml/m^2^ at 12:00-13:00, which amounted to 1066.4 ml/m^2^ after factoring-in the estimated-loss. Similarly, the cumulative recovered yield (Fig. 3-b) was 4711.1 ml/m^2^, which amounted to 5780.5 ml/m^2^ upon adding the loss factor. The comprehensive data set is affixed in Appendix A.

2. *Transient solar-to-vapor conversion efficiency*

**Fig. 5:**
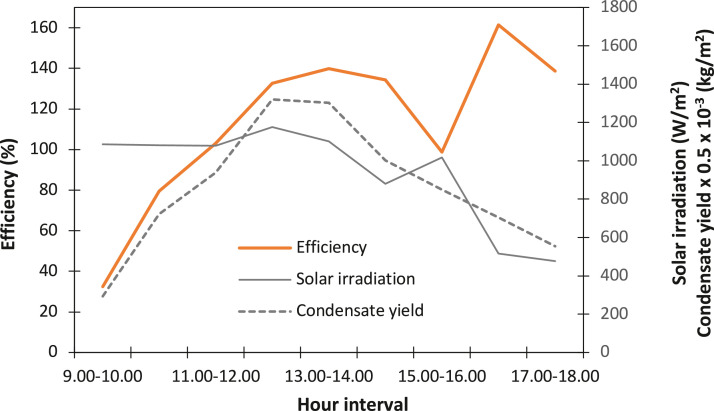
Transient solar-to-vapor conversion efficiency in relation to the instantaneous solar irradiation and the hourly condensate yield.

Evaluating the solar-to-vapor conversion efficiency as a one-off, fixed parameter on the basis of the average values of *ṁ, h_fg_* and *I_(t)_* from Table 3 gave 108.4%, which did not suggest a pattern of interaction of the solar still across the hours of insolation and the condensate yield. Whereas by evaluating the efficiency on hourly basis, the periodic hourly performance of the solar still was better understood, as shown in Fig. 5. Likewise, the trend of the instantaneous incident solar irradiation versus the hourly condensate yield became more evident. The trend hints that a rising efficiency of the solar still, as observed in the first five-hour intervals, resulted from a steady rise in condensate yield at a fairly constant solar irradiation. This is an indication of good thermal retention capacity of the solar still. Moreover, at 16:00-17:00 h, the solar irradiation declined with about 50% of the previous value, while the condensate yield only reduced with about 17% of the previous amount, giving rise to the optimum efficiency of the solar still at that interval. Meanwhile, the mean of the hourly transient efficiencies was 113.4%, showing a slight but significant variation coefficient of 0.0225 from the conventional method.

**Table 3:** Evaluation of the hourly solar-to-vapor conversion efficiency from the hourly experimental parametric values.

Hour interval	ṁ (ml/m^2^)	ṁ (kg/m^2^)	T_w_ (°C)	h_fg_ (kJ/kg)	I_(t)_ (W/m^2^)	η (%)
09.00-10.00	146.5	0.1465	40.1	2405.7	1087.3	32.4
10.00-11.00	360.5	0.3605	49.5	2383.1	1081.3	79.4
11.00-12.00	469.1	0.4691	56.8	2365.4	1077.3	103.0
12.00-13.00	660.1	0.6601	58.0	2362.5	1176.0	132.6
13.00-14.00	651.9	0.6519	57.2	2364.5	1102.3	139.8
14.00-15.00	501.2	0.5012	60.0	2357.6	880.3	134.2
15.00-16.00	424.7	0.4247	56.8	2365.4	1016.3	98.8
16.00-17.00	352.3	0.3523	56.0	2367.4	516.7	161.4
17.00-18.00	276.5	0.2765	46.0	2391.6	476.7	138.8

## Conclusions

First, we introduced in this study an approach to theoretically estimate the condensate loss in the inclined double slope solar still. We demonstrated and validated the significance and reliability of the formulated model for theoretical condensate loss estimation by showing at 15% degree of freedom a coefficient of variation of 0.117 between the actual experimental loss and the estimated value. In essence, being able to account for 18.5% loss fraction of condensate on our investigated solar still prototype translates that the reported optimum freshwater yield of 4711.1 ml/m^2^ was only recovered from 81.5% of the total condensation area. Accordingly, 5780.5 ml/m^2^ was recoverable from 100% of the condensation surface, when the loss fraction is imputed, provided other factors that may influence condensate loss are forestalled. This analysis provides a better insights into investigating the actual performance of a solar still—thus providing, eventually, a reliable conclusion for subsequent performance upgrades. Second, we also suggested and demonstrated -as a rewarding alternative to the conventional daily average efficiency- a transient solar-to-vapor conversion efficiency evaluation approach, relying on the hourly values of the experimental parameters. Thus, a coefficient of variation of 0.0225 from the comparison between the daily and average hourly-estimated solar-to-vapor conversion efficiency would be significant at stringent instances. Finally, accounting for the condensate loss -due to the slope of the condensate collection-channel- suggests an advancement in the approach to report the performance productivity of the solar still, as no study had previously accounted for condensate losses by any means whatsoever on the device.

## Recommendations

We recommend for future undertakings comprehensive investigations of other factors that potentially contribute to condensate loss in the solar still and formulation of methods to quantify such losses where outright prevention is not practical. The adoption of our proposed geometric approach for the estimation of condensate losses in the circular, tubular and parabolic type solar stills is as well suggested. By and large, a design consideration that will reduce condensate losses in the solar still to the minimum is advised.

## Declaration of Competing Interest

The authors declare that they have no known competing financial interests or personal relationships that could have appeared to influence the work reported in this paper.

## Figures and Tables

**Table:** 

Trials	Day 1		Day 2		Day 3		Average	
	m	$m_{loss}$	m	$m_{loss}$	m	$m_{loss}$	m	$m_{loss}$
09.00-10.00	0.0	-	128.4	-	311.1	-	146.5	-
10.00-11.00	409.9	-	350.6	-	321.0	-	360.5	-
11.00-12.00	414.8	-	449.4	-	543.2	-	469.1	-
12.00-13.00	498.8	-	612.3	-	869.1	-	660.1	-
13.00-14.00	577.8	-	612.3	-	765.4	-	651.9	-
14.00-15.00	454.3	-	387.7	-	661.7	-	501.2	-
15.00-16.00	375.3	-	400.0	-	498.8	-	424.7	-
16.00-17.00	237.0	-	380.2	-	439.5	-	352.3	-
17.00-18.00	261.7	-	266.7	-	301.2	-	276.5	-
Cumulative yield	3229.6	529.7	3587.7	595.6	4711.1	815.0	3842.8	657.9

## Data Availability

Data will be made available on request.

## Appendix A

Comprehensive experimental values for condensate yield (m) and recovered loss $\left(m_{loss}\right)$, in ml/m^2^, from 3-days seawater desalination experiment.
